# Autotrophic lactate production from H_2_ + CO_2_ using recombinant and fluorescent FAST-tagged *Acetobacterium woodii* strains

**DOI:** 10.1007/s00253-022-11770-z

**Published:** 2022-01-29

**Authors:** Alexander Mook, Matthias H. Beck, Jonathan P. Baker, Nigel P. Minton, Peter Dürre, Frank R. Bengelsdorf

**Affiliations:** 1 Institute of Microbiology and Biotechnology, University of Ulm, Albert-Einstein-Allee 11, 89081 Ulm, Germany; 2 Clostridia Research Group, BBSRC/EPSRC Synthetic Biology Research Centre (SBRC), University of Nottingham, Nottingham, UK

**Keywords:** *Acetobacterium woodii*, Lactate dehydrogenase, Gas fermentation, H_2_ + CO_2_, FAST, Fluorescent tag

## Abstract

Lactate has various uses as industrial platform chemical, poly-lactic acid precursor or feedstock for anaerobic co-cultivations. The aim of this study was to construct and characterise *Acetobacterium woodii* strains capable of autotrophic lactate production. Therefore, the *lctBCD* genes, encoding the native Lct dehydrogenase complex, responsible for lactate consumption, were knocked out. Subsequently, a gene encoding a d-lactate dehydrogenase (LDHD) originating from *Leuconostoc mesenteroides* was expressed in *A. woodii*, either under the control of the anhydrotetracycline-inducible promoter P_*tet*_ or under the lactose-inducible promoter P_*bgaL*_. Moreover, LDHD was N-terminally fused to the oxygen-independent fluorescence-activating and absorption-shifting tag (FAST) and expressed in respective *A. woodii* strains. Cells that produced the LDHD fusion protein were capable of lactate production of up to 18.8 mM in autotrophic batch experiments using H_2_ + CO_2_ as energy and carbon source. Furthermore, cells showed a clear and bright fluorescence during exponential growth, as well as in the stationary phase after induction, mediated by the N-terminal FAST. Flow cytometry at the single-cell level revealed phenotypic heterogeneities for cells expressing the FAST-tagged LDHD fusion protein. This study shows that FAST provides a new reporter tool to quickly analyze gene expression over the course of growth experiments of *A. woodii*. Consequently, fluorescence-based reporters allow for faster and more targeted optimization of production strains.

**Key points**

•*Autotrophic lactate production was achieved with A. woodii*.
•*FAST functions as fluorescent marker protein in A. woodii*.
•*Fluorescence measurements on single-cell level revealed population heterogeneity.*

**Supplementary Information:**

The online version contains supplementary material available at 10.1007/s00253-022-11770-z.

## Introduction

The recent and ever-developing pursuit of a clean and sustainable industry raises the need for novel, especially biological, processes that use gaseous waste streams to generate value-added products. Autotrophic fermentations employing acetogens that grow by using mixtures of CO_2_, CO and H_2_ offer a future-proof biological process, converting industrial waste gas streams to a variety of organic acids and alcohols (Bengelsdorf and Dürre 2017). CO_2_-based processes are getting more and more attractive and economically feasible, due to efforts to increase the costs of CO_2_ emission and reduce greenhouse gas emissions. One potential chemical that can be produced from CO_2_ is lactate (Eş et al. 2018). Lactate and lactic acid have a wide range of applications in food production, pharmaceutical and cosmetic products (Valli et al. 2006), and as a precursor for poly-lactic acid (PLA), which is a biodegradable plastic (Song et al. 2012). Moreover, there are also recent approaches to use lactate as feedstock in (synthetic) co-cultures to produce value-added products such as propionic acid (Selder et al. 2020) or medium-chain acids such as butyrate (Detman et al. 2019) and caproate (Liu et al. 2020). A model strain for the study of acetogens and C1 gas fermentations is *Acetobacterium woodii*. Its central metabolism (Poehlein et al. 2012) as well as its native lactate metabolism pathway, encoded by genes of the lct-operon (Weghoff et al. 2015), and the respective regulation (Schoelmerich et al. 2018), are well studied. The native lactate dehydrogenase (LDH) of *A. woodii* is coupled to electron transferring flavoprotein subunits (EtfA/EtfB) and catalyzes the oxidation of lactate to pyruvate to reduce NAD^+^ while oxidizing reduced ferredoxin Fd^2−^ in an electron-confurcating reaction (Weghoff et al. 2015). Furthermore, the genetic toolbox of *A. woodii* includes methods for gene knock-out (Schoelmerich et al. 2018; Ehsaan et al. 2016), a set of functional promoters (Beck et al. 2020), and plasmids (Hoffmeister et al. 2016). By using GusA assays, the lactose-inducible promoter P_*bgaL*_ (Hartman et al. 2011) and the anhydrotetracycline (ATc)-inducible promoter P_*tet*_ (Berens and Hillen 2003) were already characterised for *A. woodii* (Beck et al. 2020). However, such GusA assays only elucidate expression behaviour at whole-culture level. Single-cell level expression behaviour in *A. woodii* can be clarified using the fluorescence-activating and absorption-shifting tag protein (FAST) as a reporter tool. FAST is a two-component system, in which a fluorogen based on hydroxybenzylidene rhodanine (Li et al. 2017) non-covalently binds to a 14-kDa protein tag based on the photoactive yellow protein (PYP) of *Halorhodospira halophila* (Plamont et al. 2016). Currently, multiple variants of FAST such as FAST2, engineered for improved fluorescence (Tebo et al. 2018), or split-FAST, a variant to study protein–protein interactions (Tebo and Gautier 2019), are available. The FAST system was shown to be active in several prokaryotic and eukaryotic hosts (Charubin et al. 2020; Lu et al. 2021; Peron-Cane et al. 2020; Tebo et al. 2021) and as oxygen-independent reporter is well suited for in vivo measurements in anaerobic bacteria, as already demonstrated for *Clostridium acetobutylicum* (Streett et al. 2019) and *Eubacterium limosum* (Flaiz et al. 2021).

In this study, *A. woodii* was engineered to produce lactate from H_2_ + CO_2_. To ensure that lactate is not consumed by the strains, the genes *lctBCD*, encoding the native LDH/EtfAB complex, were deleted via allelic coupled exchange. The resulting deletion strain of *A. woodii* was then further engineered to produce lactate by introduction of the gene *ldhD* from *Leuconostoc mesenteroides* subsp. *mesenteroides* ATCC 8293, encoding for a d-lactate dehydrogenase (LDHD) (Li et al. 2012). Recombinant *ldhD* expression for a plasmid-based and a chromosome integration strain was controlled with the P_*tet*_ promoter. In a further step, the genes encoding for LDHD and FAST2 were codon-optimized for *A. woodii* and fused. The resulting N-terminally FAST-tagged LDHD fusion protein (NFP) was then produced in a plasmid-based *A. woodii* mutant under control of the P_*bgaL*_ promoter, to improve lactate yields and study single-cell gene expression behaviour in batch growth experiments.

## Materials and methods

### Medium and cultivation

*Escherichia coli* DH5α was used for plasmid construction and cultivated either in LB (Luria–Bertani) medium (tryptone 10 g/L, NaCl 10 g/L, yeast extract 5 g/L) (Bertani 1951) while shaking (175 rpm) or on respective agar plates at 37 °C. If necessary, LB medium was supplemented with 250 μg/mL erythromycin. *A. woodii* strains were cultivated in modified DSM 135 containing 0.20 g/L NH_4_Cl, 1.76 g/L KH_2_PO_4_, 8.44 g/L K_2_HPO_4_, 10 g/L NaHCO_3_, 0.50 g/L L-cysteine-HCl, 3 g/L yeast extract, 2 mL/L SL9 trace element solution (Tschech and Pfennig 1984), 2 mL/L vitamin solution (Wolin et al. 1963),1 mL/L %(w/v) selenite-tungstate solution (Tschech and Pfennig 1984), 1 mL/L resazurin (1 g/L) and 20 μg/mL uracil. The medium was prepared anaerobically by sevenfold exchange of the atmosphere with CO_2_ + N_2_ (20:80). After autoclaving, the medium was supplemented with 0.33 g/L MgSO_4_ • 7 H_2_O and, if needed, with 5 μg/mL clarithromycin or 20 μg/mL thiamphenicol. Autotrophic cultivations of *A. woodii* strains were performed in 50 mL medium in rubber-sealed 500-mL Müller-Krempel flasks, gassed with H_2_ + CO_2_ (67:33) to an overpressure of 110 kPa. When, during cultivation, overpressure had fallen below 50 kPa due to gas, the flasks were gassed again to 110 kPa. Growth experiments were started with respective seeds of strains from DMSO stock cultures, which were then cultivated in 5 mL DSM 135 mod. in hungates. Cells from these 5-mL seed cultures were transferred to 50-mL medium in Müller-Krempel flasks with the aforementioned gas atmosphere for adaptation. The gas-adapted cells were then used as inoculum for autotrophic growth experiments. If needed, induction of gene expression was conducted by supplementation of the culture broth with 6.8 g/L lactose in case of the P_*bgaL*_ promoter or 300 ng/mL anhydrotetracycline (ATc) in case of the P_*tet*_ promoter.

### Strain and plasmid construction

The *A. woodii* strains used in this study (Table 1) are based on the parental strain *A. woodii ∆lctBCD ∆pyrE*. This strain is a gene knock-out variant of *A. woodii* DSMZ 1030 engineered via *pyrE*-linked allelic coupled exchange (Ehsaan et al. 2016). The *A. woodii ∆pyrE* strain contains a deleted *pyrE* (orotate phosphoribosyl transferase) coding gene which is used as counterselection marker for further deletions or additions to the chromosome (Ehsaan et al. 2016). This technique was used to delete *lctBCD*, three genes of *A. woodii*’s native *lct*-operon encoding lactate dehydrogenase coupled to electron transferring subunits. Plasmids used in this work are listed in Table 2. Construction of the plasmids pJIR750 and pJIR750_P_*tet*__ldhD_LM, as well as the respective strains, has been described earlier by Beck (2020). Furthermore, a detailed description of chromosome integration steps leading *A. woodii* P_*tet*_*_ldhD*_CI_ is given there. Construction of the plasmid [pMTL83251_P_*bgaL*__FAST], carrying the FAST-encoding gene *feg*, is described by Flaiz et al. (2021). For the construction of the plasmid [pMTL83251_P_*bgaL*__NFP], the sequences of FAST2 (*feg2*) and the lactate dehydrogenase gene *ldhD* of *Leuconostoc mesenteroides* (*ldhD*) were codon-optimized for *A. woodii* and synthetized by Eurofins Genomics (Eurofins Genomics GmbH, Luxemburg) (see Figure S1). The resulting fragment *feg2_awo_opt* (GenBank-Nr. OL439951) was amplified via PCR with primers N_FAST2_awo_opt_fwd and N_FAST2_awo_opt_rev. The fragment *ldhD_awo_opt* (GenBank-Nr. OL439952) was amplified via PCR with primers N_ldhD_awo_opt_fwd and N_ldhD_awo_opt_rev. The sequences of the primers are shown in Table 3. By aforementioned PCR amplifications, 20-bp overlaps between the two fragments and the backbone as well as a GGGGS linker (GGTGGTGGTGGTTCT) were introduced. The backbone pMTL83251_P_*bgaL*__FAST (Flaiz et al. 2021) was digested with *Bam*HI and *Xho*I to excise the FAST fragment and generate overlaps to the aforementioned codon-optimized fragments. The backbone and fragments were ligated using the NEBuilder® HiFi DNA Assembly Kit (New England Biolabs, Ipswich, Ma, USA), resulting in the plasmid pMTL83251_P_*bgaL*__NFP, carrying the fusion gene *NFP_awo_opt* (GenBank-Nr. OL439953). The parental strain *A. woodii ∆lctBCD ∆pyrE* was transformed with the plasmids by electroporation. Preparation of electrocompetent *A. woodii* cells and electroporation were performed as described previously (Hoffmeister et al. 2016). The strains were verified by restriction analysis and sequencing of either the plasmids isolated from *A. woodii* or *E. coli* DH5α, which was also used as host strain for plasmid construction.

**Table 1:** Bacterial strains

Strain	Genotype	Description	Source
*E. coli* DH5α	*Escherichia coli* DH5α	Used for plasmid construction	Thermo Fisher Scientific Inc., Waltham, MA, USA
*A. woodii ∆lctBCD ∆pyrE*	*Acetobacterium woodii ∆lctBCD ∆pyrE*	Uracil dependent *A. woodii* DSM 1030 mutant with deleted lactate dehydrogenase complex	This work
*A. woodii* [pJIR]	*Acetobacterium woodii ∆lctBCD ∆pyrE* [pJIR]	*A. woodii* mutant carrying the empty backbone plasmid pJIR750	Beck(2020)
*A. woodii* [P_*tet*_*_ldhD*]	*Acetobacterium woodii ∆lctBCD ∆pyrE* [pJIR_P_*tet*__ldhD]	*A. woodii* mutant expressing *ldhD* under control of the P_*tet*_ promoter	Beck (2020)
*A. woodii* P_*tet*_*_ldhD*_CI_	*Acetobacterium woodii ∆lctBCD ∆pyrE::pyrE_*P_*tet*_*_ldhD*	*A. woodii* chromosome integration mutant with reconstituted *pyrE*, expressing *ldhD* under control of the P_*tet*_ promoter	Beck (2020)
*A. woodii* [p83]	*Acetobacterium woodii ΔlctBCD ΔpyrE* [pMTL83251]	*A. woodii* mutant carrying the empty backbone plasmid pMTL83251	This work
*A. woodii* [P_*bgaL*_*_*FAST]	*Acetobacterium woodii ΔlctBCD ΔpyrE* [pMTL83251_P_*bgaL*__FAST]	*A. woodii* mutant expressing *feg* under control of the P_*bgaL*_ promoter	This work
*A. woodii* [P_*bgaL*_*_ldhD_*NFP]	*Acetobacterium woodii ΔlctBCD ΔpyrE* [pMTL83251_P_*bgaL*__NFP]	*A. woodii* mutant expressing the codon-optimized *feg2*-*ldhD* fusion gene under control of the P_*bgaL*_ promoter	This work

**Table 2:** Plasmids and their relevant features

Plasmid	Relevant features	Source
pJIR750	Cm^r^, ColE1 ori^−^, *lacZ,* pIP404 ori^+^, MCS	Bannam and Rood (1993)
pJIR750_P_*tet*__ldhD	As pJIR750, P_*tet*_, *ldhD* (LEUM_1756, *L. mesenteroides* subsp. *Mesenteroides* ATCC 8293)	Beck (2020)
pMTL83251	*ermB*, ColE1 ori^−^, pCB102^+^, *traJ*, *lacZ*, MCS	Heap et al. (2009)
pMTL83251_P_*bgaL*__FAST	As pMTL83251, P_*bgaL*_, *feg*	Flaiz et al. (2021)
pMTL83251_P_*bgaL*__NFP	As pMTL83251, P_*bgaL*_, optimized *feg2-ldhD (NFP_awo_opt, GenBank-Nr.:* OL439953*)*	This work
pEX-A258-ldhD_Awo_opt	*ampR*, pUC ori, *ldhD_awo_opt* (*GenBank-Nr.:* OL439952*)*	Eurofins Genomics GmbH, Luxemburg
pEX-A128-FAST2_Awo_opt	*ampR*, pUC ori, *feg2_awo_opt*(*GenBank-Nr.:* OL439951)	Eurofins Genomics GmbH, Luxemburg

**Table 3:** Primers used for plasmid construction

Primer	Sequence (5ʹ ➔ 3ʹ)
N_FAST2_awo_opt_fwd	tta aat gta ttg gga ggg tgg atc cat gga aca cgt tgc tg
N_FAST2_awo_opt_rev	caa aga tct tag aac cac cac cac cta ccc gtt tga caa ata cc
N_ldhD_awo_opt_fwd	caa acg ggt agg tgg tgg tgg ttc taa gat ctt tgc tta tgg c
N_ldhD_awo_opt_rev	agc ttg cat gtc tgc agg cct cga gtt aat att caa ccg caa ttg

### Analytics

During growth experiments, up to 2-mL samples were drawn from the culture bottles to measure OD_600_, pH and metabolic end products. Pressure in the bottles was measured by a manometer before sampling, to calculate gas uptake. The GENESYS 30 vis spectrophotometer (Thermo Fisher Scientific Inc., Waltham, MA, USA) was used for determination of OD_600_. For determination of fermentation products, the samples were centrifuged at 17,968 × *g* at 4 °C for 20 min to pelletize cells. One half of the supernatant was used for determination of pH via a pH probe, and the other half was used to analyze fermentation products via high-performance liquid chromatography (HPLC).

The concentrations of acetate and lactate in the culture supernatant were determined using the Agilent 1260 Infinity II HPLC system (Agilent Technologies, Santa Clara, CA, USA), with a diode array detector and a refractive index detector. For analysis, 20 μL of supernatant was injected onto a polystyrene divinylbenzene copolymer packed 150- × 8-mm column (CS-Chromatogaphie-Service GmbH, Langerwehe, Germany). The system was operated at 40 °C with 5 mM H_2_SO_4_ as mobile phase with a flow rate of 0.7 mL/min. Data analysis was performed with the OpenLAB CDS ChemStation Edition A.01.03 software (Agilent Technologies, Santa Clara, CA, USA).

### Fluorescence determination

Fluorescence during growth experiments with *A. woodii* [P_*bgaL*_*_*FAST] and *A. woodii* [P_*bgaL*_*_ldhD_*NFP] was measured using a microplate reader and flow cytometry. Two milliliters of culture broth was harvested for each sampling point and centrifuged at 7,711 × *g* for 10 min at 4 °C. The supernatant was discarded and the cell pellet suspended in 2 mL PBS (137 mM NaCl, 2.7 mM KCl, 10 mM Na_2_HPO_4_, 1.8 mM KH_2_PO_4_) with pH 7.4. The cell suspension was centrifuged again at 7,711 × *g* for 10 min at 4 °C and suspended in 1 mL PBS. The suspension was diluted to OD_600_ of 1 for further measurements.

The SYNERGY H1 microplate reader (BioTek, Bad Friedrichshall, Germany) was used for fluorescence measurements at whole-culture level. One hundred microliters of cell suspension was transferred to 96-well black flat bottom microtitre plates (Greiner Bio-One GmbH, Frickenhausen, Germany). Shortly before measurements, 10 μL of ^TF^Lime (The Twinkle Factory, Paris, France) was added to the respective wells. For measurements, the wells were individually excited at *λ*_ex_ = 480 nm and emissions were detected at *λ*_em_ = 541 nm. Obtained values were normalised against the OD_600_ of the used cell suspension.

Fluorescence at single-cell level was measured via the Amnis® CellStream® flow cytometer (Luminex Corporation, Austin, TX, USA). The PBS washed cell suspension was further diluted to an OD_600_ of 0.01 and supplemented with ^TF^Lime to a final concentration of 10 μM. The fluorescence of the cell suspension was measured using an excitation wavelength *λ*_ex_ = 488 nm and detected with a 528/46 nm emission filter. A minimum of 10,000 events were recorded and analyzed with the CellStreamTM Analysis tool (Version 1.2.152, Luminex Corporation, Austin, TX, USA). The following gating strategy was used to differentiate between fluorescent and non-fluorescent subpopulations based on measured events. Events measured without addition of ^TF^Lime were gated as negative. Events with fluorescent signal caused by addition of ^TF^Lime and located outside of the negative gate were gated positive. To prevent double gating in nearby subpopulations a small gap was left between gates termed n.d. (not determined).

## Results

### Autotrophic growth of recombinant lactate producing *A. woodii*

**Fig. 1:**
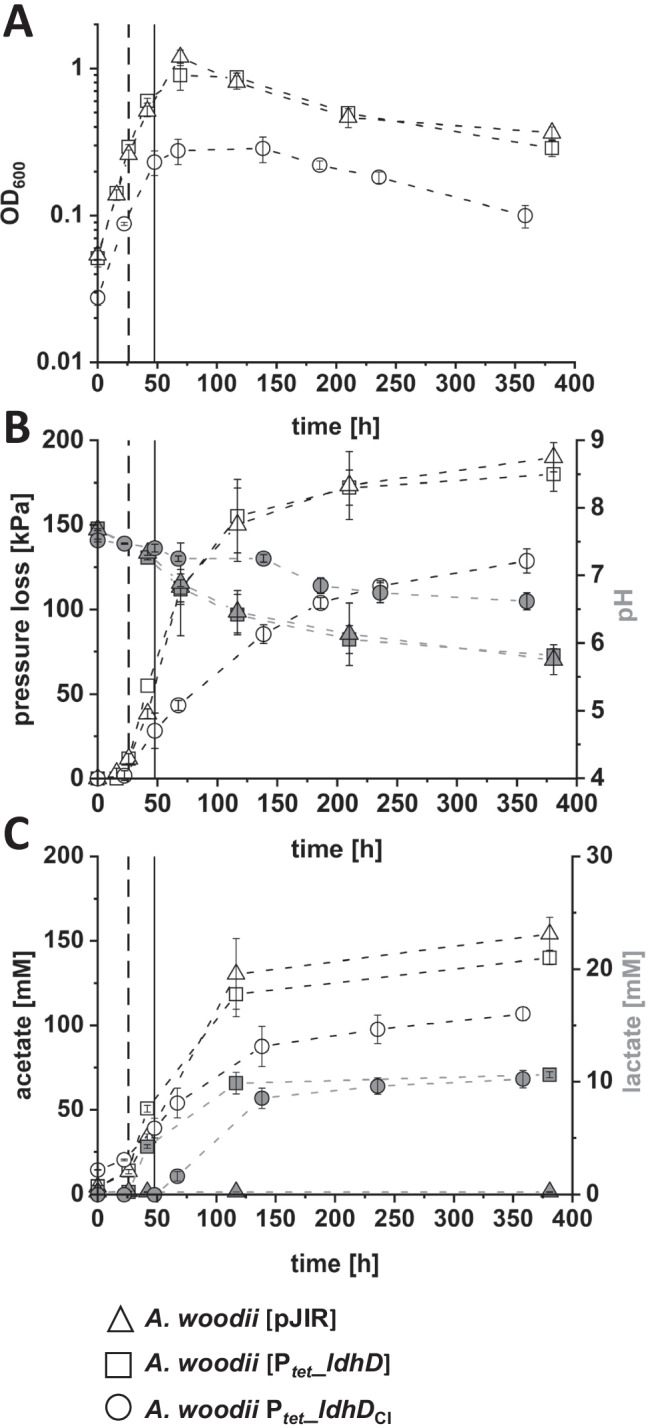
Autotrophic lactate production by recombinant *A. woodii* strains. Optical density (**A**); pH, accumulated pressure loss (**B**); and product spectrum (**C**) of autotrophic fermentation of *A. woodii* [pJIR] (△), *A. woodii* [P_*tet*__*ldhD*] (□) and *A. woodii* P_*tet*_*_ldhD*_CI_ (○) cultivated in modified DSMZ 135 medium in bottles with 110 kPa H_2_ + CO_2_ in the headspace. White symbols refer to the left *y*-axis, grey symbols to the respective right *y*-axis. Time of induction is indicated with the dashed vertical line for *A. woodii* [P_*tet*__*ldhD*] and with a solid vertical line for *A. woodii* P_*tet*_*_ldhD*_CI_. Error bars represent the standard deviation of biological triplicates

The strains used in this study are based on *A. woodii ∆lctBCD ∆pyrE*, a uracil-dependent mutant of *A. woodii* DSM 1030, which was constructed to eliminate lactate consumption by deletion of the *lctBCD* genes. The constructed and verified strains *A. woodii* [pJIR], *A. woodii* [P_*tet*_*_ldhD*] and *A. woodii* P_*tet*_*_ldhD*_CI_ were cultivated autotrophically with H_2_ + CO_2_ as energy and carbon source (Fig. 1). After recombinant *ldhD* gene expression was induced by ATc, the latter two strains produced up to 10 mM lactate under autotrophic growth conditions. The growth rates of the control strain *A. woodii* [pJIR] (*μ* = 0.17 1/h) and the plasmid-based lactate-producing strain *A. woodii* [P_*tet*_*_ldhD*] (*μ* = 0.14–0.15 1/h) were not affected after induction and reached peak optical densities (OD_600_) of 1.19 and 0.89, respectively (Fig. 1A). In contrast, the growth rate for *A. woodii* P_*tet*_*_ldhD*_CI_ was much lower after induction (Table 4) and a peak OD_600_ of only 0.28 was reached. Throughout the fermentation, the accumulated pressure loss sums up to 190 kPa, indicating consumption of H_2_ + CO_2_ by *A. woodii* [pJIR] and *A. woodii* [P_*tet*_*_ldhD*]. The pH of the culture broth of these two cultures decreased from 7.7 to 5.8 (Fig. 1B). During 358 h of cultivation of the chromosome integration mutant *A. woodii* P_*tet*_*_ldhD*_CI_, a total pressure loss of 129 kPa was calculated, while the pH only dropped from 7.5 to 6.6 in the same time. *A. woodii* [pJIR] and *A. woodii* [P_*tet*_*_ldhD*] produced acetate peak concentrations of 153 mM and 140 mM, respectively (Fig. 1C). *A. woodii* P_*tet*_*_ldhD*_CI_ reached a peak acetate concentration of only 102 mM. Both *A. woodii* [P_*tet*_*_ldhD*] and *A. woodii* P_*tet*_*_ldhD*_CI_ were able to produce about 10 mM lactate, despite differences in growth (Fig. 1C). The lactate production rate (0.1 mM/h) after induction with ATc for both strains was similar (Table 4). For *A. woodii* [P_*tet*_*_ldhD*], a lactate/acetate ratio of 0.12 could be determined, while *A. woodii* P_*tet*_*_ldhD*_CI_ showed a respective ratio of 0.15.

**Table 4:** Growth rates, acetate and lactate production rates. Data was obtained before and after induction of recombinant *A. woodii* strains in 50-mL batch cultures. Growth and acetate production rates are given before and after induction, lactate production rates only after induction

**Strain**	***μ* [1/h]**	***μ***_**post induction**_** [1/h]**	**r**_**acetate**_** [mM/h]** pre/post induction	**r**_**lactate**_** [mM/h]**	**Lactate/acetate ratio**
*A. woodii* [pJIR]	0.017	-	0.35/1.29	-	-
*A. woodii* [P_*tet*__*ldhD*]	0.015	0.014	0.36/1.07	0.1	0.12
*A. woodii* P_*tet*_*_ldhD*_CI_	0.006	0.001	0.27/0.52	0.1	0.15
*A. woodii* [p83]	0.017	-	1.65/2.15	-	-
*A. woodii* [P_*bgaL*_*_*FAST]	0.015	0.020	1.64/2.19	0.004	-
*A. woodii* [P_*bgaL*_*_ldhD_*NFP]	0.016	0.003	1.82/0.87	0.37	0.33

### Autotrophic growth experiments with fluorescent lactate producers

**Fig. 2:**
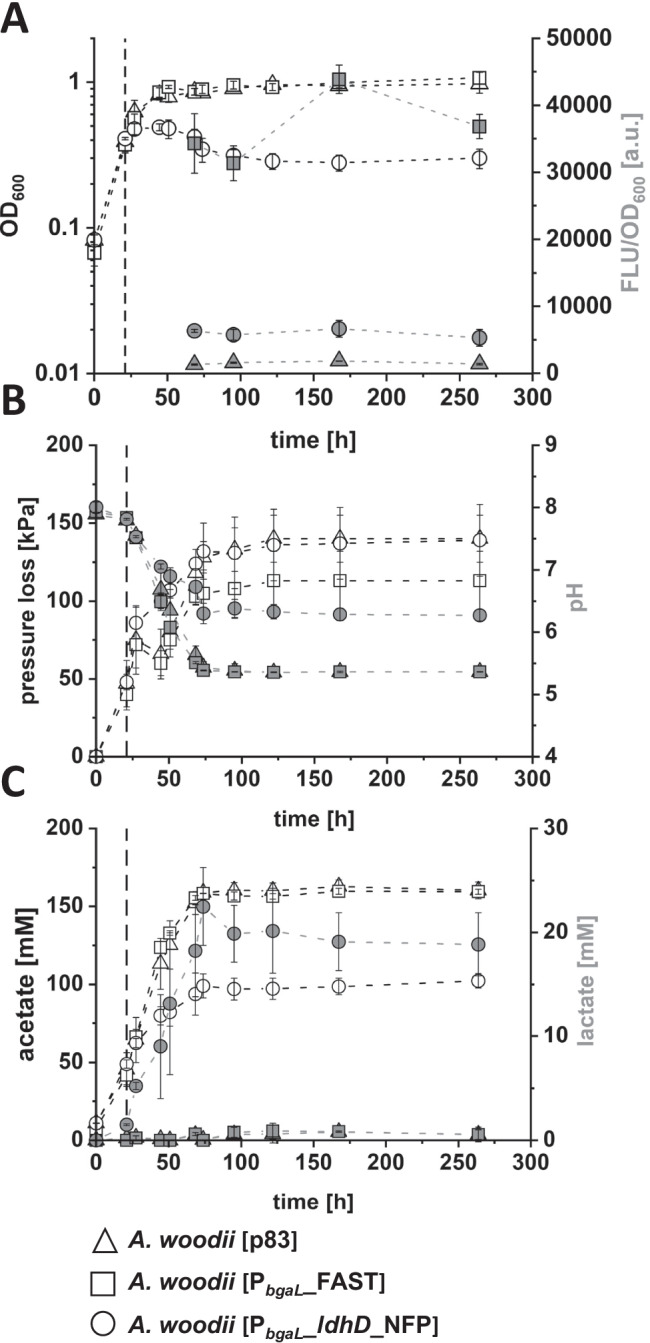
FAST-tagged LDHD production yields maximal autotrophic lactate production. Optical density, fluorescence (**A**); pH, accumulated pressure (**B**); and product spectrum (**C**) of autotrophic fermentation of *A. woodii* [p83] (△), *A. woodii* [P_*bgaL*_*_*FAST] (□) and *A. woodii* [P_*bgaL*_*_ldhD_*NFP] (○) cultivated in modified DSMZ 135 medium in bottles with 110 kPa H_2_ + CO_2_ in the headspace. White symbols refer to the left y-axis, grey symbols to the respective right y-axis. Time of induction for the P_*bgaL*_-carrying strains is indicated with the dashed vertical line. Fluorescence readouts were normalised to the OD_600_ of used cell suspensions. Error bars represent standard deviation of biological triplicates

Furthermore, a recombinant *A. woodii* strain was constructed to produce a codon-optimized LDHD N-terminally fused to a codon-optimized fluorescent FAST tag, resulting in the respective fusion protein NFP. ATc was found to be photoactive in the VIS range of 400 to 500 nm, leading to spectral overlaps with the green fluorescent fluorogen ^TF^Lime (*λ*_Em,max_ = 541). Therefore, the P_*tet*_ promoter was exchanged with the lactose-inducible P_*bgaL*_ promoter to facilitate clear fluorescence signals as well as lactate production. The newly constructed strain *A. woodii* [P_*bgaL*_*_ldhD_*NFP] was genetically verified and cultivated in an autotrophic growth experiment together with *A. woodii* [P_*bgaL*_*_*FAST] (produces solely FAST controlled by P_*bgaL*_) and *A. woodii* [p83] (carrying the empty plasmid) as control strains. Expression of FAST and NFP encoding genes was induced by addition of lactose (21 h after inoculation) and resulted in production of 18.8 mM lactate as well as bright fluorescence mediated by FAST. The growth rate of the lactate-producing strain *A. woodii* [P_*bgaL*_*_ldhD_*NFP] (*μ* = 0.16 1/h) was severely decreased after induction of gene expression (Table 4). Consequently, the strain reached a peak OD_600_ of only 0.49 shortly after induction and decreased to 0.3 at the end of cultivation (Fig. 2A). Fluorescence measurements over time confirmed stable production of the N-terminally tagged FAST-LDHD fusion protein (Fig. 2A). *A. woodii* [P_*bgaL*_*_ldhD_*NFP] showed a 3.6-fold higher fluorescence compared to the non-fluorescent *A. woodii* [p83]. The *feg* expressing control strain *A. woodii* [P_*bgaL*_*_*FAST] exhibited increased fluorescence around 24-fold higher than the non-fluorescent *A. woodii* [p83] control strain. During cultivation, the pressure loss of up to 139 kPa, reveals H_2_ + CO_2_ consumption by the individual strains (Fig. 2B). The pH of the culture broth dropped from 8 to 6.3 for *A. woodii* [P_*bgaL*_*_ldhD_*NFP] and to 5.4 for the other two strains (Fig. 2B). Final concentration of acetate was 102 mM for *A. woodii* [P_*bgaL*_*_ldhD_*NFP] and 160 mM for both control strains. *A. woodii* [P_*bgaL*_*_ldhD_*NFP] reached a final lactate concentration of 18.8 mM (Fig. 2C), with a lactate production rate of 0.37 mM/h. The lactate/acetate ratio of 0.33 recorded for *A. woodii* [P_*bgaL*_*_ldhD_*NFP] (Table 4) was 2.2–2.8-fold higher than those of the comparable P_*tet*_ controlled lactate-producing *A. woodii* [P_*tet*_*_ldhD*] and *A. woodii* P_*tet*_*_ldhD*_CI_ strains.

### Fluorescence response upon induction of gene expression in lactate producing *A. woodii*

**Fig. 3:**
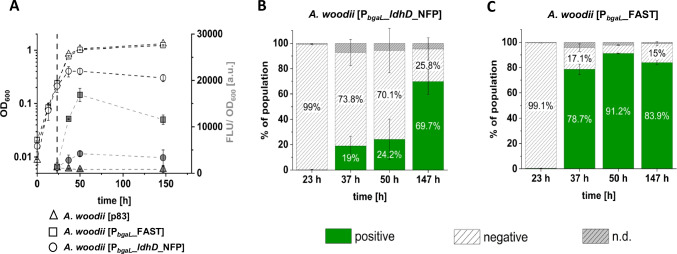
Heterogeneous *A. woodii* populations detected by FAST-mediated fluorescence. Growth and fluorescence of the strains *A. woodii* [p83], *A. woodii* [P_*bgaL*_*_*FAST] and *A. woodii* [P_*bgaL*_*_ldhD_*NFP] during autotrophic batch fermentation in bottles with modified DSMZ 135 medium with 110 kPa H_2_ + CO_2_ overpressure. Fluorescence was measured on whole-culture level via a microplate-reader (grey symbols), in addition to determination of OD_600_ (white symbols) (**A**). Fluorescence on single-cell level (**B** and **C**) was determined via flow cytometry. Fluorescence was activated via addition of 5 μM ^TF^Lime. **A** The dashed line indicates the time of induction. **B**, **C** Columns represent percentages of subpopulations inside the fluorescence positive or negative gates, while n.d. (not determined) was calculated as remaining fraction of non-gated events. Error bars represent the standard deviation of biological triplicates

The FAST-tagged LDHD fusion Protein (NFP) was used to monitor fluorescence of cultures on total population level via a microplate reader (Fig. 3A). Furthermore, dynamic changes of fluorescence at single-cell level were followed using a flow cytometer (Fig. 3B–C). Therefore, the strains *A. woodii* [P_*bgaL*_*_ldhD_*NFP], *A. woodii* [P_*bgaL*_*_*FAST] and *A. woodii* [p83] were cultivated in batch experiments as described before. Again, shortly after induction, the growth of *A. woodii* [P_*bgaL*_*_ldhD_*NFP] stopped, while the two other strains grew unimpeded (Fig. 3A) leading to a fourfold lower OD_600_ of *A. woodii* [P_*bgaL*_*_ldhD_*NFP] compared to the control strains at the end of the fermentation. The fluorescence measurements by plate reader showed a 21-fold higher fluorescence of *A. woodii* [P_*bgaL*_*_*FAST] and fivefold higher fluorescence of *A. woodii* [P_*bgaL*_*_ldhD_*NFP] compared to *A. woodii* [p83]. Measurements with a flow cytometer revealed that 27 h after induction, already 91.2% of the *A. woodii* [P_*bgaL*_*_*FAST] population were fluorescent (Fig. 3C). In contrast, single-cell measurements of *A. woodii* [P_*bgaL*_*_ldhD_*NFP] cultures resulted in 24.2% of fluorescent cells in the same period (Fig. 3B). Interestingly, the percentage of fluorescent cells for *A. woodii* [P_*bgaL*_*_ldhD_*NFP] cultures increased slowly when compared to *A. woodii* [P_*bgaL*_*_*FAST], from 19 to 24% after 50 h of cultivation and up to 70% after 147 h (Fig. 3B).

## Discussion

Three recombinant lactate-producing *A. woodii* strains were investigated containing either the native LDHD from *L. mesenteroides* or a codon-optimized FAST-tagged version of the lactate dehydrogenase. The P_*tet*_ controlled lactate-producing strains *A. woodii* [P_*tet*_*_ldhD*] and *A. woodii* P_*tet*_*_ldhD*_CI_ were induced by ATc, which is a derivate of tetracycline and should have reduced antibiotic activity at the applied concentration (Gossen and Bujard 1993). Moreover, ATc is also known to be photoactive in the spectrum between 400 and 500 nm (Almedia et al. 1997). Thus, P_*tet*_ was replaced with the lactose-inducible P_*bgaL*_ promoter, which was described as leaky but ten times stronger by using GusA assays (Beck et al. 2020). In consequence, *A. woodii* [P_*bgaL*_*_ldhD_*NFP] is not dependent on ATc for induction, therefore enabling FAST-mediated fluorescence measurements with the green fluorescent fluorogen ^TF^Lime. Furthermore, the promoter exchange presumably increased expression of the NFP coding gene. Additionally, the LDHD and FAST2 coding genes were codon-optimized and fused resulting in the *feg2*-*ldhD* gene, because codon usage can determine protein production levels (Liu et al. 2021). A further change was the switch of replicons from pIP404 (pJIR750) to pCB102 (pMTL83251), which possibly has an influence on plasmid stability, copy number and transformation efficiency (Yu et al. 2012), and in consequence could positively influence the productivity of the constructed strains (Hoffmeister et al. 2016). Highest autotrophic lactate productivity was achieved with the strains presented in this study (final titre of 1.7 g/L in 11 days for *A. woodii* [P_*bgaL*_*_ldhD_*NFP]) compared to earlier metabolic engineering attempts. A photoautotrophic mutant strain of *Synechocystis* sp. PCC 6803 was able to produce a final titre of 1.14 g/L d-lactate over the course of 24 days (Varman et al. 2013), and an engineered *Rhodococcus opacus* mutant produced 146 mg/L lactate during 300 h of autotrophic cultivation (Li et al. 2015) respectively. However, autotrophic lactate production is still far behind in comparison to heterotrophic processes. For example, Iwasaki et al. (2017) engineered *Moorella thermoacetica* to produce 40 mM lactate from fructose. Baek et al. (2017) engineered a *Saccharomyces cerevisiae* strain expressing *ldhD* from *L. mesenteroides* to produce up to 82.6 g/L lactic acid in a fed-batch fermentation with glucose as feedstock. Nevertheless, CO_2_-based fermentation processes can be expected to receive increasing attention, when considering the current efforts towards carbon–neutral feedstocks (Halder et al. 2019).

An important factor in terms of anaerobic autotrophic fermentation is the energy balance of the used biocatalyst (Schuchmann and Müller 2014), especially, if novel heterologous pathways are expressed to produce new products (Molitor et al. 2017). The central metabolism of *A. woodii* depends on the connection of the Wood-Ljungdahl pathway (WLP) (Poehlein et al. 2012) and chemiosmotic energy conservation processes mediated by a [FeFe]-hydrogenase (HydABCD) (Schuchmann and Müller 2012), a ferredoxin:NAD + oxidoreductase (Rnf) complex and an ATP synthase (Hess et al. 2013). The interplay between these enzymes results in 0.3 ATP for each mol of acetate produced (Müller et al. 2018). *A. woodii*’*s* native lactate dehydrogenase complex (Ldh/EtfAB), encoded by the *lctBCD* genes, enables lactate oxidation coupled to electron confurcation (Weghoff et al. 2015). Respective genes were deleted from strains used in this study, to facilitate lactate production without its consumption by *A. woodii*.

**Fig. 4:**
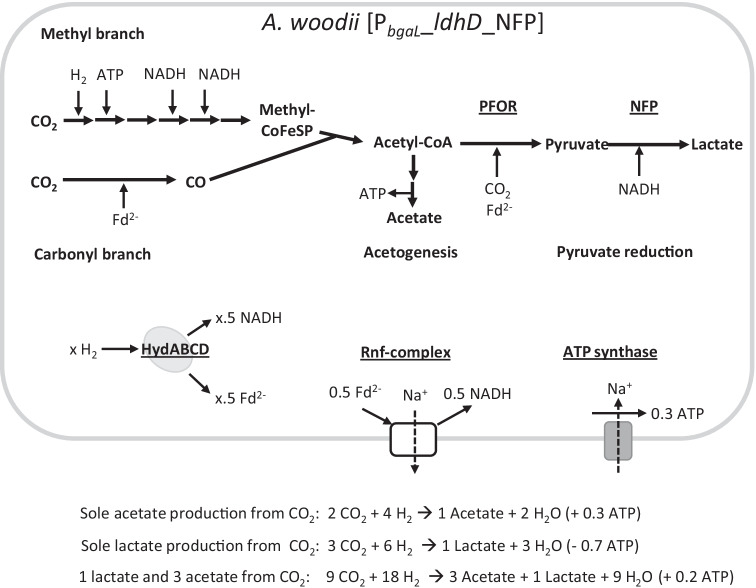
Schematic overview of central *A. woodii* [P_*bgaL*_*_ldhD*_NFP] metabolism. Simplified reaction scheme for the Wood-Ljungdahl pathway (WLP) split into carboxyl and methyl branches as well as acetogenesis. Furthermore, the pyruvate reduction pathway is including a NADH-dependent lactate dehydrogenase. Chemiosmotic energy conservation is achieved by interplay between [FeFe]-hydrogenase (HydABCD), ferredoxin:NAD^+^ oxidoreductase (Rnf) complex and ATP synthase. Ferredoxin is depicted in its reduced state (Fd^2−^). CoFeSP, corrinoid iron-sulphur protein; PFOR, pyruvate:ferredoxin oxidoreductase; NFP, FAST N-terminally fused to d-lactate dehydrogenase; *x*, representing different values that vary depending on the overall reaction. Overall reaction equations are given for sole acetate, sole lactate production and a lactate/acetate ratio of 1/3

The LDHD from *Leuconostoc mesenteroides* is NADH-dependent and favours pyruvate reduction over lactate oxidation in the pH range from 6 to 8 in situ (Li et al. 2012). Referring to the central metabolism combined with the recombinant lactate production pathway (Fig. 4), it is evident that there are strong energy balance constraints to consider. Compared to native autotrophic acetogenesis, one additional mol each of CO_2_, NADH and Fd^2−^ are needed for lactate production. CO_2_ and reduction equivalents are supplied through WLP and HydABCD from the H_2_ + CO_2_ gas phase. However, the ATP-demanding step in the methyl branch leading to acetyl-CoA has a large impact on lactate production. The ATP invested here can only be obtained by the acetogenic autotrophic metabolism. If lactate were the sole end product, 0.3 ATP could still be generated through the ATP synthase while the invested ATP from the methyl branch cannot be balanced through acetogenesis, yielding a net loss of 0.7 ATP per mol acetyl-CoA and subsequently lactate, as calculated before (Bertsch and Müller 2015). From these calculations alone, total conversion of CO_2_ to lactate is not feasible. If, however, 3 mol of acetate is produced by the combination of WLP and acetogenesis, formally, 0.9 ATP can be provided. This surplus potentially allows a fourth acetyl-CoA to be converted to lactate, with HydABCD providing reduction equivalents (NADH, Fd^2−^) and an additional CO_2_ from the gas phase. The remaining 0.2 ATP are then available for further metabolism and maintenance. With this theoretical pathway for lactate production, the resulting low energy regime seems to be a reasonable explanation for the abrupt inhibition of growth after induction of gene expression in *A. woodii* [P_*bgaL*_*_ldhD_*NFP]. Furthermore, the resulting theoretical lactate/acetate ratio of 0.33 is well met by the optimized lactate-producing strain *A. woodii* [P_*bgaL*_*_ldhD_*NFP] grown with H_2_ + CO_2_.

Curiously, the P_*tet*_ controlled lactate producers *A. woodii* [P_*tet*_*_ldhD*] and *A. woodii* P_*tet*_*_ldhD*_CI_ exhibited different behaviour. While both reached lactate/acetate ratios of 0.12 and 0.15, respectively, only the chromosome integration mutant *A. woodii* P_*tet*_*_ldhD*_CI_ exhibited a growth stop after induction of gene expression and consequently lactate production. However, despite the growth stop, *A. woodii* P_*tet*_*_ldhD*_CI_ reached similar lactate concentrations of 10 mM with only a third of the OD_600_ as *A. woodii* [P_*tet*_*_ldhD*]. This phenomenon implies that the single-cell productivity of the chromosome integration strain was higher than that of the plasmid-based producing strain, which can be explained in two basic premises. Firstly, the change from possibly multiple copies of the pJIR750 plasmid to a single integration site on the bacterial chromosome reduces the burden on the cells by lowering the demand for metabolic and gene expression resources (Borkowski et al. 2016). Simply the removal of the antibiotic resistance *catP* gene encoded on the pJIR750 backbone, in theory, frees up acetyl-CoA which otherwise would be used for acetylation of thiamphenicol (Syriopoulou et al. 1981). Secondly, the growth behaviour of *A. woodii* [P_*tet*_*_ldhD*], which was similar to the empty backbone strain even after induction heavily implies heterogeneities within the lactate-producing culture. The calculated growth rates for the *A. woodii* [P_*tet*_*_ldhD*] strain could possibly stem from a subpopulation of non-lactate-producing, fast-growing cells overshadowing the growth stop of the lactate-producing subpopulation (Mustafi et al. 2014).

A first step to elucidate possible heterogeneities within the lactate-producing strains was using the FAST-tagged fluorescent NFP protein, allowing to discriminate between protein-producing and non-producing cells. FAST was already established as fluorescent marker in various anaerobic bacteria (Streett et al. 2019; Flaiz et al. 2021) and its independence from oxygen (Plamont et al. 2016) renders it an excellent choice for monitoring gene expression in acetogens (Flaiz et al. 2021). Comparing the results for gene expression of *feg* to the *feg2*-*ldhD* fusion gene (Fig. 3) shortly after induction, distinct expression behaviour was noticeable for both strains. The high shares of fluorescent cells in flow cytometry measurements correlate well with the results on whole-culture level for *A. woodii* [P_*bgaL*_*_*FAST] obtained with the plate reader. For *A. woodii* [P_*bgaL*_*_ldhD_*NFP], the response upon induction of gene expression is slower than for *A. woodii* [P_*bgaL*_*_*FAST] and results in a lower fraction of fluorescent cells. Furthermore, heterogeneities between biological replicates were recorded (cp. large standard deviation in Fig. 3). The growth stop of *A. woodii* [P_*bgaL*_*_ldhD_*NFP] after induction implies that despite only 24.2% of cells recognized as green fluorescent, most of the cells should produce lactate. Otherwise, the non-lactate-producing subpopulation of *A. woodii* [P_*bgaL*_*_ldhD_*NFP] would be expected to grow similarly to the empty backbone control strain A*. woodii* [p83]. Thus, it has to be assumed that the FAST-tagged LDHD protein NFP exhibits different fluorescence than the FAST protein itself. LDHD in solution is tetrameric (Li et al. 2012). An influence of multimer formation on the accessibility and conformation of FAST binding pockets cannot be ruled out, even though the N-terminal tagging serves to point the tag away from the sites of aggregation around the core of LDHD (Figure S2). The hypothetical fluorogenic binding site of the PYP-derived FAST2 was proposed to employ tyrosine and glutamic acid for the formation of hydrogen bonds, similar to the 4-hydroxycinnamic acid binding site of PYP (Tebo et al. 2018). Changes in distance of these residues to each other or surrounding residues forming the binding pocket could strongly influence the fluorescence strength when fluorogen is sub-optimally bound. For further investigations in this regard, crystal structures of the NFP components or NFP itself would be needed.

Another factor influencing population heterogeneity could be the lactose-inducible promoter P_*bgaL*_ itself. The P_*bgaL*_ promoter was initially used in *Clostridium perfringens* (Hartman et al. 2011) and since then tested in *C. acetobutylicum* (Al-Hinai et al. 2012), *C. ljungdahlii* (Banerjee et al. 2014), *A. woodii* (Beck et al. 2020) and *E. limosum* (Flaiz et al. 2021). Most studies focus on whole-population marker protein assays such as GusA assays or on product titres of recombinant strains to ascertain the functionality of P_*bgaL*_. However, a close look at the published data provides a strong indication towards cell-to-cell variance, as shown through microscopic images (Hartman et al. 2011), standard deviations between biological replicates (Al-Hinai et al. 2012; Beck et al. 2020) or FAST-based cytometry (Flaiz et al. 2021).

Autotrophic lactate production employing acetogens, as demonstrated in this study, is a possible way to reduce CO_2_ emissions and transform this greenhouse gas into value-added products. The main problem is the low energy yield that acetogens have to deal with, which could be addressed by changing fermentation conditions. *A. woodii* is known to grow not only with H_2_ + CO_2_, but also with syngas (H_2_ + CO_2_ + CO) (Novak et al. 2021), methanol (Kremp et al. 2018) or formate (Moon et al. 2021), substrates performing better with respect to energy conservation and ATP yields. Especially the combination of C1 substrates and their co-utilization is a promising strategy to improve energy conversion of acetogens (Neuendorf et al. 2021). Furthermore, the heterogeneity of the high lactate-producing *A. woodii* [P_*bgaL*_*_ldhD_*NFP] strain needs further investigation to ascertain the cause for phenotypic variations during lactate production.

## Supplementary Information

Below is the link to the electronic supplementary material.

Supplementary file1 (PDF 351 KB)

## Data Availability

The datasets generated during and/or analysed during the current study are available from the corresponding author on reasonable request.
