# Supplementary material for: Autotrophic lactate production from H2 + CO2 using recombinant and fluorescent FAST-tagged Acetobacterium woodii strains
Source: Appl Microbiol Biotechnol. 2022 Jan 29;106(4):1447–58. doi: 10.1007/s00253-022-11770-z (PMC8882112; doi:10.1007/s00253-022-11770-z)
Supplement: Supplementary file 1 — Supplementary file1 (PDF 351 KB) [file 253_2022_11770_MOESM1_ESM.pdf]

Supplementary Materials

Applied Microbiology and Biotechnology

Autotrophic lactate production from H_2 + CO_2 using recombinant and fluorescent FAST-tagged *Acetobacterium woodii* strains

Authors

[1]Alexander Mook[†], [1]Matthias H. Beck[†], [2]Jonathan P. Baker, [2]Nigel P. Minton, [1]Peter Dürre, [1]Frank R. Bengelsdorf*

[1]Institute of Microbiology and Biotechnology, University of Ulm, Albert-Einstein-Allee 11, 89081 Ulm, Germany.

[2]Clostridia Research Group, BBSRC/EPSRC Synthetic Biology Research Centre (SBRC), University of Nottingham, Nottingham, UK.

[†]Contributed equally

*Corresponding author: Frank R. Bengelsdorf

e-mail address: frank.bengelsdorf@uni-ulm.de

telephone: (0049)731-50-22715

fax numbers (0049)731-50-22719

Supplement 1: DNA sequences of codon optimised *feg2*, *ldhD* and the fusion gene encoding for NFP. Sequence for the GGGGS-linker is underlined

>feg2_awo_opt (GenBank-Nr.: OL439951)

ATGGAACACGTTGCGTTTGGTAGCGAGGACATTGAGAACACGTTAGCAAAGATGGATGACGGCCAGCTGGACGGCCTTGCTTTTGGGGCCAT
ACAACTTGATGGCGATGGAAACATCTTGCAATACAATGCAGCCGAAGGCGATATAACAGGTAGAGATCCGAAACAAGTTATCGGGAAAAATTT
CTTCAAGGATGTGGCTCCTGGGACTGATTCGCCAGAATTCTATGGAAAGTTTAAAGAAGGTGTGGCGTCTGGAAATTTAAATACCATGTTTGAA
TGGATGATTCCAACATCCCGAGGTCCCACCAAAGTTAAAATTCATATGAAAAAAGCATTGAGTGGAGATTCATATTGGGTATTTGTCAAACGGG
TATAA

>ldhD_awo_opt (GenBank-Nr.: OL439952)

ATGAAGATCTTTGCTTATGGCATTAGAGACGACGAAAAACCCTCCTTAGAAGAATGGAAAGCCGCCAACCCAGAAATCGAAGTGGACTACACC
CAAGAGCTACTTACCCCTGAGACCGCTAAGCTGGCCGAGGGCAGCGATTCGGCCGTGGTCTACCAGCAGCTTGACTATACTAGGGAAACCTT
GACAGCTCTCGCGAACGTTGGTGTTACCAACTTGTCTCTTCGCAATGTAGGAACTGACAACATTGACTTTGATGCGGCCCGGGAGTTTAATTTT
AACATCTCAAACGTGCCTGTTTATTCTCCAAACGCTATTGCAGAGCACTCAATGATACAGTTGTCACGCTTATTACGTCGAACTAAAGCACTAG
ATGCGAAAATTGCTAAACACGACTTGCGTTGGGCTCCAACGATTGGTCGAGAAATGAGAATGCAAACAGTTGGCGTTATTGGGACGGGCCATA
TAGGAAGAGTCGCCATAAATATCTTGAAAGGCTTCGGGGCCAAAGTGATAGCATACGATAAATACCCAAATGCAGAACTTCAAGCTGAGGGTT
TGTATGTGGATACACTCGATGAACTGTATGCACAAGCAGATGCAATAAGTTTGTATGTACCGGGTGTTCCGGAAAATCATCATCTGATCAATGC
TGATGCGATCGCTAAGATGAAGGATGGAGTTGTAATAATGAATGCAGCTAGGGGTAATCTTATGGATATAGATGCGATTATCGATGGGTTAAAT
TCCGGGAAGATTAGTGATTTCGGAATGGATGTCTATGAAAATGAAGTAGGACTGTTTAATGAAGATTGGTCGGGAAAAGAATTTCCCGATGCC
AAAATTGCGGATTTAATTGCACGGGAAAATGTATTAGTTACACCGCATACAGCCTTTTATACTACGAAAGCGGTCTTAGAAATGGTTCATCAAA
GCTTTGATGCCGCAGTCGCATTCGCTAAAGGTGAAAAACCGGCAATTGCGGTTGAATATTAA

>NFP_awo_opt (GenBank-Nr.: OL439953)

ATGGAACACGTTGCGTTTGGTAGCGAGGACATTGAGAACACGTTAGCAAAGATGGATGACGGCCAGCTGGACGGCCTTGCTTTTGGGGCCAT
ACAACTTGATGGCGATGGAAACATCTTGCAATACAATGCAGCCGAAGGCGATATAACAGGTAGAGATCCGAAACAAGTTATCGGGAAAAATTT
CTTCAAGGATGTGGCTCCTGGGACTGATTCGCCAGAATTCTATGGAAAGTTTAAAGAAGGTGTGGCGTCTGGAAATTTAAATACCATGTTTGAA
TGGATGATTCCAACATCCCGAGGTCCCACCAAAGTTAAAATTCATATGAAAAAAGCATTGAGTGGAGATTCATATTGGGTATTTGTCAAACGGG
TA<u>GGTGGTGGTGGTTCT</u>AAGATCTTTGCTTATGGCATTAGAGACGACGAAAAACCCTCCTTAGAAGAATGGAAAGCCGCCAACCCAGAAATCG
AAGTGGACTACACCCAAGAGCTACTTACCCCTGAGACCGCTAAGCTGGCCGAGGGCAGCGATTCGGCCGTGGTCTACCAGCAGCTTGACTAT
ACTAGGGAAACCTTGACAGCTCTCGCGAACGTTGGTGTTACCAACTTGTCTCTTCGCAATGTAGGAACTGACAACATTGACTTTGATGCGGCC
CGGGAGTTTAATTTTAACATCTCAAACGTGCCTGTTTATTCTCCAAACGCTATTGCAGAGCACTCAATGATACAGTTGTCACGCTTATTACGTCG
AACTAAAGCACTAGATGCGAAAATTGCTAAACACGACTTGCGTTGGGCTCCAACGATTGGTCGAGAAATGAGAATGCAAACAGTTGGCGTTAT
TGGGACGGGCCATATAGGAAGAGTCGCCATAAATATCTTGAAAGGCTTCGGGGCCAAAGTGATAGCATACGATAAATACCCAAATGCAGAACT
TCAAGCTGAGGGTTTGTATGTGGATACACTCGATGAACTGTATGCACAAGCAGATGCAATAAGTTTGTATGTACCGGGTGTTCCGGAAAATCAT
CATCTGATCAATGCTGATGCGATCGCTAAGATGAAGGATGGAGTTGTAATAATGAATGCAGCTAGGGGTAATCTTATGGATATAGATGCGATTA
TCGATGGGTTAAATTCCGGGAAGATTAGTGATTTCGGAATGGATGTCTATGAAAATGAAGTAGGACTGTTTAATGAAGATTGGTCGGGAAAAGA
ATTTCCCGATGCCAAAATTGCGGATTTAATTGCACGGGAAAATGTATTAGTTACACCGCATACAGCCTTTTATACTACGAAAGCGGTCTTAGAA
ATGGTTCATCAAAGCTTTGATGCCGCAGTCGCATTCGCTAAAGGTGAAAAACCGGCAATTGCGGTTGAATATTAA

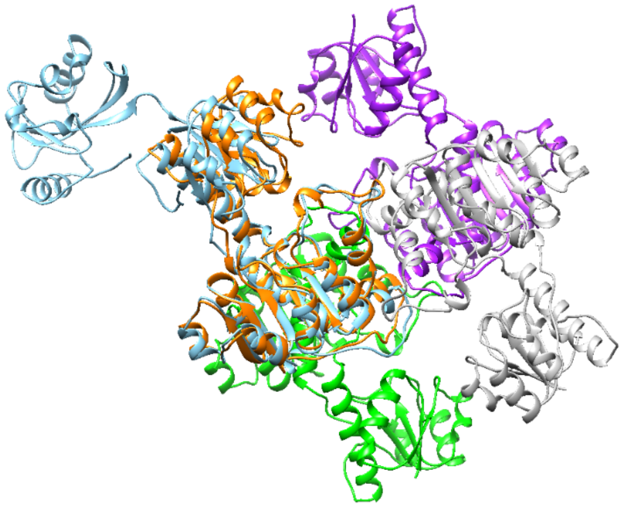

Supplement 2: Predicted tertiary structure of NFP superimposed on a tetramer of D-lactate dehydrogenase from *Pseudomonas aeruginosa*. Orange, green, purple and gray chains depict the singular chains of the *P. aeruginosa* D-lactate dehydrogenase as tetramer (PDB: 3wwz.1; GMQE 0.55; QSQE 0.41, sequence identity 0.36). Light blue NFP chain was modelled with roseTTAfold (Baek et al. 2021) (confidence 0.79) and superimposed on the PDB model with the MatchMaker in UCSF Chimera (pettersen et al. 2004). For MatchMaker the Needleman-Wunsch alignment algorithm was used. Light blue outlier depicts FAST2 fused with a GGGS linker to the N-terminal end of the LDHD chain.

References:

Baek M, DiMaio F, Anishchenko I, Dauparas J, Ovchinnikov S, Lee GR, Wang J, Cong Q, Kinch LN, Schaeffer RD, Millán C, Park H, Adams C, Glassman CR, DeGiovanni A, Pereira JH, Rodrigues AV, van Dijk AA, Ebrecht AC, Opperman DJ, Sagmeister T, Buhlheller C, Pavkov-Keller T, Rathinaswamy MK, Dalwadi U, Yip CK, Burke JE, Garcia KC, Grishin NV, Adams PD, Read RJ, Baker D (2021) Accurate prediction of protein structures and interactions using a three-track neural network. Sci 373:871–876. doi: 10.1126/science.abj8754

Pettersen EF, Goddard TD, Huang CC, Couch GS, Greenblatt DM, Meng EC, Ferrin TE (2004) UCSF Chimera--a visualization system for exploratory research and analysis. J Comput Chem 25(13):1605-1612. doi: 10.1002/jcc.20084
